# Supplementary material for: Multilevel predictors categorization for post-CABG atrial fibrillation prediction
Source: Biol Methods Protoc. 2025 Dec 12;11(1):bpaf092. doi: 10.1093/biomethods/bpaf092 (PMC12791823; doi:10.1093/biomethods/bpaf092)
Supplement: bpaf092_Supplementary_Data [file bpaf092_supplementary_data.docx]

# Supplemental Material

**Appendix A.**

Clinical and functional characteristics of patients with coronary artery disease

| **Predictor** | **Group PoAF (n=280)** | | **Group without PoAF (n=1025)** | | **OR [95% CI]** | **p-value** |
| --- | --- | --- | --- | --- | --- | --- |
|  | Ме (Q1; Q3)/abs | Mean ± SD/% | Ме (Q1; Q3) | Mean ± SD |  |  |
| Age, years | 66 (61; 71) | 65.7 ±  6.9 | 63 (58; 69) | 62.9 ± 7.78 | - | <0.000001 |
| Female, abs. (%) | 76 | 27.14% | 237 (23.12%) |  | 1.24 [0.917; 1.673] | 0.188 |
| Height, cm | 170 (165; 176) | 170.1 ± 8.2 | 170 (165; 176) | 170  ± 8 | - | 0.802 |
| Weight, kg | 80 (73; 90) | 82.5 ± 14 | 80 (73; 90) | 82.1 ± 13 | - | 0.86 |
| BMI, ${kg/m}^{2}$ | 27.9 (25.4; 31.2) | 28.56± 4.85 | 28.1 (25.8; 31) | 28.51±4.69 | - | 0.27 |
| LVEF, % | 60 (54; 64) | 57.6 ±8.5 | 60 (51; 66) | 57.4 ±9.5 | - | 0.89 |
| RLVMI, c. u. | 1.01 (0.86; 1.18) | 1.05 ±0.307 | 0.98 (0.84; 1.16) | 1.02 ±0.264 | - | 0.263 |
| RTI, c. u. | 0.408 (0.363; 0.455) | 0.415 ±0.083 | 0.417 (0.37; 0.458) | 0.42 ±0.088 | - | 0.442 |
| LV ESD, cm | 3.3 (3.2; 3.7) | 3.49 ±0.58 | 3.35 (3; 3.8) | 3.45 ±0.59 | - | 0.185 |
| LV EDD, cm | 5.1 (4.8; 5.425) | 5.15  ± 0.58 | 5.1 (4.7; 5.4) | 5.1  ± 0.56 | - | 0.284 |
| Systolic pressure gradient Ao/LV, mm Hg | 7 (5; 9) | 8.67 ± 7.34 | 6 (5; 8) | 7.95 ± 7.88 | - | 0.051 |
| MPAP, mm Hg | 25 (23; 30) | 27.8 ± 8.97 | 25 (23; 30) | 26.9 ± 7.77 | - | 0.221 |
| LAL, cm | 3.9 (3.6; 4.2) | 3.93±0.56 | 3.9 (3.6; 4.3) | 3.98 ± 0.6 | - | 0.339 |
| LAD, cm | 4.6 (4; 5.1) | 4.57±0.74 | 4.3 (3.8; 4.9) | 4.38±0.73 | - | 0.0002 |
| Indexed LA volume, ml/m2 | 33.3 (24.5; 43.1) | 33.69±17.9 | 30.2 (23.7; 39.3) | 32.21±16.4 | - | 0.125 |
| RAL, cm | 3.8 (3.5; 4.1) | 3.8±0.5 | 3.7 (3.4; 4) | 3.71±0.52 | - | 0.0023 |
| RAD, cm | 4.5 (4.1; 4.9) | 4.5±0.64 | 4.3 (3.8; 4.8) | 4.28±0.66 | - | 0.0000033 |
| P, ms | 100 (100; 100) | 102.44±8.5 | 100 (100; 100) | 101.99±8 | - | 0.69 |
| PQ, ms | 160 (140; 180) | 161.3±32.7 | 150 (140; 180) | 156.8±36.7 | - | 0.025 |
| QRS, ms | 80 (80; 100) | 88.97± 14.97 | 80 (80; 100) | 94.4± 19.29 | - | 0.00036 |
| RR, ms | 950 (895; 1090) | 961.8±166 | 900 (800; 1080) | 943.7±166.5 | - | 0.129 |
| QT, ms | 400 (360; 430) | 396.8±34.7 | 380 (360; 420) | 387.9±38.5 | - | 0.00143 |
| Creatinine, µmol/l | 92.71 (79; 110) | 95.3±25.7 | 97 (83; 110) | 98.7±24.2 | - | 0.036 |
| GFR, ml/min | 158.8 (135.1; 188.7) | 171.57±54.2 | 162.8 (135.1; 190.9) | 166.54±26.1 | - | 0.43 |
| CHF III-IV FC, abs. (%) | 43 (15.36%) |  | 124 (12.1%) |  | 1.56 [0.76; 1.47] | 0.169 |
| History of MI, abs. (%) | 32 (18.5%) |  | 143 (20%) |  | 0.91 [0.6; 1.4] | 0.75 |
| Stable angina pectoris III-IV FC | 65 (34.6%) |  | 247 (37.6%) |  | 1.14 [0.81; 1.61] | 0.478 |
| Extracardiac arteriopathy | 96 (34.3%) |  | 343 (33.46%) |  | 1.04 [0.79; 1.38] | 0.85 |
| AH, abs. (%) | 225 (80.35%) |  | 813 (79.31%) |  | 1.7 [0.81; 3.7] | 0.81 |
| Aortic stenosis, abs. (%) | 6 (2.1%) |  | 20 (1.95%) |  | 1.24 [0.49; 3.15] | 0.74 |
| TR, abs. (%) | 52 (18.57%) |  | 112(10.93%) |  | 1.8 [1.26; 2.6] | 0.0009 |
| MR, abs. (%) | 89 (31.78%) |  | 296 (28.88%) |  | 1.13 [0.85; 1.51] | 0.38 |
| AR, abs. (%) | 20 (7.15%) |  | 67 (6.5%) |  | 1.12 [0.66; 1.88] | 0.82 |
| CKD, abs. (%) | 75 (26.78%) |  | 262 (25.56%) |  | 1.07 [0.79; 1.43] | 0.74 |
| COPD, abs. (%) | 45 (16.07%) |  | 137 (13.36%) |  | 1.2 [0.72; 1.95] | 0.29 |
| DM, abs. (%) | 68 (24.28%) |  | 257 (25.07%) |  | 0.95 [0.7; 1.3] | 0.82 |
| Previous stroke, abs. (%) | 27 (9.64%) |  | 89 (8.68%) |  | 1.2 [0.71; 1.76] | 0.70 |
| Hemoglobin, g/l | 142 (131; 152) | 141±16.6 | 143(131; 153) | 141±16.9 | - | 0.79 |
| Red blood cells,10^12^/l | 4.69 (4.31; 5.03) | 4.65±0.58 | 4.69 (4.30; 5.06) | 4.65±0.58 | - | 0.97 |
| Leukocytes,10^9^/l | 6.8 (5.7; 8) | 7± 2 | 6.9 (5.8; 8.37) | 7.2±2.2 | - | 0.175 |
| Lymphocytes,10^9^/l | 1.87 (1.44; 2.42) | 1.9±0.73 | 2 (1.53; 2.53) | 2.1±0.92 | - | 0.09 |
| Platelets,10^9^/l | 228 (182; 266) | 225±57 | 232 (192; 273) | 237±65 | - | 0.0336 |
| Total cholesterol,mmol/l | 4.35 (3.66; 5.4) | 4.6±1.36 | 4.45 (3.7; 5.43) | 4.7±1.41 | - | 0.15 |
| Glucose, mmol/l | 5.63 (5.11; 6.21) | 6±1.7 | 5.67 (5.13; 6.52) | 6.2±1.8 | - | 0.144 |
| Total protein, g/l | 70.9 (66.3; 73.7) | 69.6±7.3 | 71.4 (67.9; 75.2) | 71.1±7.8 | - | 0.004 |
| Total bilirubin, µmol/l | 16.96 (12.1; 23.813) | 19.3±10 | 16.3 (11.7; 22.95) | 18.8±10.2 | - | 0.338 |
| Triglycerides, mmol/l | 1.48 (1.16; 1.88) | 1.68±1.1 | 1.63 (1.2; 2.2) | 1.82±0.94 | - | 0.003 |
| Urea, mmol/l | 6 (5; 7.13) | 6.37±1.94 | 6 (4.97; 7.35) | 6.47±2.29 | - | 0.954 |
| Thrombin time, s | 19.6 (16.6; 21.5) | 20±6.14 | 19.9 (17.1; 21.4) | 20.1±7.2 | - | 0.67 |
| PTI, % | 93.3 (86; 99.6) | 92±14.58 | 94 (86.88; 102) | 94.26±25.3 | - | 0.148 |
| INR | 1.05 (1; 1.13) | 1.12±0.55 | 1.03 (0.98; 1.1) | 1.06±0.124 | - | 0.073 |
| SBP, mm Hg | 130 (130;150) | 137±21 | 130 (125; 140) | 135±20 | - | 0.0229 |
| DBP, mm Hg | 80 (75; 80) | 80±8 | 80 (70; 80) | 79±8.5 | - | 0.00775 |
| Heart rate, beats/min | 68 (62; 75) | 70±12 | 68 (62; 72) | 69±10 | - | 0.178 |

**Abbreviations:** CI - confidence interval, BMI - body mass index, LV - left ventricle, LVEF - LV ejection fraction, RLVMI - relative left ventricular myocardial mass index, RTI - relative thickness index of left ventricle posterior wall, LV ESD - end systolic dimension, LV EDD - end diastolic dimension, MPAP - mean pulmonary artery pressure, LAL - left atrium medial-lateral size, LAD - left atrium anterior-posterior size, RAL - right atrium longitudinal size, RAD - right atrium transverse size, GFR - glomerular filtration rate, CHF - Congestive Heart Failure, FC- functional class, AH - arterial hypertension, TR - Tricuspid regurgitation, MR - mitral regurgitation, AR - aortic regurgitation , CKD - Chronic kidney disease, COPD- chronic obstructive pulmonary disease, DM - diabetes mellitus, PTI - prothrombin time index, INR- international normalized ratio, SBP - systolic blood pressure, DBP - diastolic blood pressure.

**Appendix B.**

Weighting coefficients in a multivariate logistic regression model without predictors standardization

| **Predictors** | **Weight coefficients** |
| --- | --- |
| Age | 0.036538 |
| RAD | 0.476447 |
| TR | 0.643446 |
| QRS | -33.214139 |
| QT | 12.580338 |
| RR | 0.551974 |
| PQ | 1.767283 |
| P | 14.158973 |
| ESD | 0.140284 |
| Intercept | -10.54463818 |

**Abbreviations:** RAD - right atrium transverse size, TR - Tricuspid regurgitation
